# Supplementary material for: Identifying Site-Specific Superoxide and Hydrogen Peroxide Production Rates From the Mitochondrial Electron Transport System Using a Computational Strategy
Source: Function (Oxf). 2021 Sep 20;2(6):zqab050. doi: 10.1093/function/zqab050 (PMC8788716; doi:10.1093/function/zqab050)
Supplement: zqab050_Supplementary_Files [file zqab050_supplementary_files.zip › ETS-ROS_Model_Supplement_vrev.docx]

**Supplement for**

**Identifying Site-specific Superoxide and Hydrogen Peroxide Production Rates from the Mitochondrial Electron Transport System Using a Computational Strategy**

Quynh V. Duong1, Yan Levitsky2, Maria J. Dessinger2, Jasiel O. Strubbe-Rivera3, Jason N. Bazil2, *

*1Department of Biochemistry and Molecular Biology, Michigan State University, East Lansing, Michigan, United States*

*2Department of Physiology, Michigan State University, East Lansing, United States*

*3Department of Pharmacology and Toxicology, Michigan State University, East Lansing, United States*

*Corresponding author: Jason N. Bazil

Email: [jnbazil@msu.edu](mailto:jnbazil@msu.edu)

**Running title**: Cardiac electron transport system sources of free radical production

**Keywords**: Electron transport system (ETS), mitochondria, reactive oxygen species, enzyme kinetics, oxidative stress, computational biology, ischemia/reperfusion injury, forward electron transport, reverse electron transport

**ETS-ROS Model Description**

The present model is extended from an earlier model of mitochondrial bioenergetics1. The calcium handling processes are retained for convenience but not used in the model simulations presented in the accompanying article. External calcium was removed with excess EGTA in the experiments analyzed with the model. Several flux expressions and model parameters were updated based on the recent works of Bazil et al.2-5, as laid out in this model supplemental material section. This portion of the supplement consists of four sections. Section 1 (S1) lists all the state variables comprising the model, dissociation constants, and other physiochemical parameters. S2 introduces the set of 22 non-linear ODEs, four algebraic conservation expressions (for mitochondrial ATP, NADH, QH2, and inter-membrane space c2+), and ten non-linear cation ODEs (mitochondrial and extra-mitochondrial H+, Na+, K+, Mg2+ and Ca2+). S3 presents the model rate equations and the associated parameter definitions and values. S4 includes addition figures and analysis described in the accompanying article. S5 describes the code used to simulate the model and generate the plots given in the paper. Refer to computer code for exact model details as there may be unintentional minor translational errors in the equations below.

The outer-mitochondrial membrane (OMM) is highly permeable to ions, metabolites, and substrates of low molecular weight under our experimental conditions. As such, all permeable inter-membrane space (IMS) state variables were replaced with their respective extra-mitochondrial counterparts, and thus not included in the model. This has the benefit of making the system of non-linear ODEs less stiff, contain fewer state variables, and shortens simulation time without significantly altering the simulation results. The only IMS state variables explicitly simulated in the model are oxidized and reduced forms of cytochrome c (c3+ and c2+).

**S1 - Model State Variables, Dissociation Constants, and Physiochemical and General Parameter Values**

The state variables used in the model, their definitions, and their units are provided in Table S1.1. All the dissociation constants used in the model are presented in Table S1.2. Dissociation constants are corrected for appropriate temperature (T = 37 oC) and ionic strength (I = 0.17 M), corresponding to the experimental conditions. The physiochemical and general model parameters are presented in Table S1.3.

| **Table S1.1.** Model State Variables | | |
| --- | --- | --- |
| State Variable | Definition | Units |
| ΔΨ | Mitochondrial membrane potential | mV |
| *Mitochondrial State Variables* | | |
| [H+]m | Mitochondrial free proton concentration | M |
| [Na+]m | Mitochondrial free sodium concentration | M |
| [K+]m | Mitochondrial free potassium concentration | M |
| [Mg2+]m | Mitochondrial free magnesium concentration | M |
| [Ca2+]m | Mitochondrial free calcium concentration | M |
| [ATP]m | Total mitochondrial ATP concentration | M |
| [ADP]m | Total mitochondrial ADP concentration | M |
| [Pi]m | Total mitochondrial Pi concentration | M |
| [NADH]m | Total mitochondrial NADH concentration | M |
| [NAD]m | Total mitochondrial NAD concentration | M |
| [QH2]m | Total mitochondrial ubiquinol concentration | M |
| [Q]m | Total mitochondrial ubiquinone concentration | M |
| [O2.-]m | Total mitochondrial superoxide concentration | M |
| [H2O2]m | Total mitochondrial hydrogen peroxide concentration | M |
| [CaPi]m | Calcium phosphate precipitate content | M |
| *Intermembrane Space (IMS) State Variables* | | |
| [c2+]i | Total IMS cytochrome c2+ (reduced) concentration | M |
| [c3+]i | Total IMS cytochrome c3+ (oxidized) concentration | M |
| *Extra-Mitochondrial State Variables* | | |
| [H+]e | Extra-mitochondrial free proton concentration | M |
| [Na+]e | Extra-mitochondrial free sodium concentration | M |
| [K+]e | Extra-mitochondrial free potassium concentration | M |
| [Mg2+]e | Extra-mitochondrial free magnesium concentration | M |
| [Ca2+]e | Extra-mitochondrial free calcium concentration | M |
| [ATP]e | Total extra-mitochondrial ATP concentration | M |
| [ADP]e | Total extra-mitochondrial ADP concentration | M |
| [Pi]e | Total extra-mitochondrial Pi concentration | M |
| [O2.-]e | Total extra-mitochondrial superoxide concentration | M |
| [H2O2]e | Total extra-mitochondrial hydrogen peroxide concentration | M |
| [Reso]e | Total resorufin concentration |  |

| **Table S1.2.** Dissociation Constants at 37 ºC, I = 0.17 M | | | |
| --- | --- | --- | --- |
| Parameter | Definition | Value | Reference |
|  | Proton ATP binding constant | 10-6.57 M | 6 |
|  | Sodium ATP binding constant | 10-1.17 M | 6 |
|  | Potassium ATP binding constant | 10-1.03 M | 6 |
|  | Magnesium ATP binding constant | 10-3.99 M | 6 |
|  | Calcium ATP binding constant | 10-3.97 M | 6 |
|  | Proton ADP binding constant | 10-6.39 M | 6 |
|  | Sodium ADP binding constant | 10-1.01 M | 6 |
|  | Potassium ADP binding constant | 10-0.90 M | 6 |
|  | Magnesium ADP binding constant | 10-3.08 M | 6 |
|  | Calcium ADP binding constant | 10-2.87 M | 6 |
|  | Proton Pi binding constant | 10-6.68 M | 6 |
|  | Sodium Pi binding constant | 10-0.63 M | 6 |
|  | Potassium Pi binding constant | 10-0.43 M | 7 |
|  | Magnesium Pi binding constant | 10-1.51 M | 8 |
|  | Calcium Pi binding constant | 10-2.31 M | 8 |
|  | 1st proton EGTA binding constant | 10-9.60 M | 9 |
|  | 2nd proton EGTA binding constant | 10-8.97 M | 9 |
|  | Potassium EGTA binding constant | 10-1.24 M | 9 |
|  | Magnesium EGTA binding constant | 10-5.24 M | 9 |
|  | Calcium EGTA binding constant | 10-11.0 M | 9 |

| **Table S1.3.** Physiochemical and General Model Parameters | | | |
| --- | --- | --- | --- |
| Parameter | Definition | Value | Reference |
| *R* | Ideal gas constant | 8.314x10-3 kJ/K/mol | physical constant |
| *T* | Temperature | 310.15 K | - |
| *F* | Faraday’s constant | 96.487x10-3 kJ/mV/mol | physical constant |
| *Cmito* | IMM capacitance | 1.45x10-3 nmol/mg/mV | 10 |
| *Ntot* | Total NAD concentration | 3 mM | 11 |
| *Qtot* | Total Q concentration | 20 mM | 2 |
| *Ctot* | Total Cyt C concentration | 200 µM | 2 |
| *Atot* | Total AdN concentration | 10 mM | 11 |
| *Volx* | Matrix H2O volume fraction | 1 µl/mg | a |
| *Voli* | IMS H2O volume fraction | 0.11 µl/mg | a |
| *Voll* | IMM Lipid volume fraction | 0.29 µl/mg | 5 |
| *Vole* | buffer H2O volume fraction | 10 ml/mg | b |
| [*EGTA*] | Extra-mitochondrial EGTA concentration | 1 mM | c |
| Abbreviations: IMM, inner mitochondrial membrane; NAD, nicotinamide adenine dinucleotide; Q, ubiquinone; Cyt C, cytochrome c; AdN, adenine nucleotide.  a Approximate mitochondrial matrix and IMS volumes.  b Based on mitochondrial concentration of 0.1 mg/ml.  c Based on experimental conditions. | | | |

**S2 - Model Differential-Algebraic Equations**

Model Differential Equations (Bioenergetics)

*Mitochondrial Membrane Potential:*

|  | (2.1) |
| --- | --- |

*Mitochondrial State Variables:*

|  | (2.2) |
| --- | --- |
|  | (2.3) |
|  | (2.4) |
|  | (2.5) |
|  | (2.6) |
|  | (2.7) |
|  | (2.8) |
|  | (2.9) |
|  | (2.10) |
|  | (2.11) |
|  | (2.12) |
|  | (2.13) |

*Inter-Membrane Space (IMS) State Variables*:

|  |  |
| --- | --- |

*Extra-Mitochondrial State Variables:*

|  | (2.14) |
| --- | --- |
|  | (2.15) |
|  | (2.16) |
|  | (2.17) |
|  | (2.18) |
|  | (2.19) |
|  | (2.20) |
|  | (2.21) |
|  | (2.22) |

Mitochondrial Conservation Algebraic Equations

The mitochondrial species for adenine nucleotides (AdNs), nicotinamide adenine dinucleotides (NADH), ubiquinone (QH2), and reduced cytochrome c (c2+) are conserved in the model. The conservation is implemented by using an algebraic expression to govern the conservation. This is done using a mass matrix with the integration algorithm and setting the appropriate rows equal to a zero-row vector.

|  | (2.23) |
| --- | --- |
|  | (2.24) |
|  | (2.25) |
|  | (2.26) |

Model Differential Equations (Cations)

The cation differential equations for the mitochondrial and extra-mitochondrial compartments are derived using the method outlined in Vinnakota et al.12. The general method will be presented with the understanding that compartment specific concentrations and transport rates are to be used where appropriate. In some cases, these compartment specific equations will be given. Due to the large expressions resulting from the derivation, the method used to obtain them is presented versus explicitly showing all the terms that enter the differential equations. The equations used in the model are obtained by solving the linear system of equations given in Equation 2.27.

The generalized system of equations relating the cation differential equations is

|  | (2.27) |
| --- | --- |

where is the partial derivative of the concentration of *bound* X with respect to Y (Y can equal X) andis the flux of X into or out of the compartment.

Assuming higher order cation binding is negligible, the partial derivative expressions are defined below where *Nr* is the number of reactants, *Li* is the *i*th ligand (such as ATP, ADP, and Pi), is the dissociation constant for the *i*th ligand and *j*th cation couple and *Pi* is the binding polynomial for the *i*th ligand as originally defined by Alberty 13.

|  | (2.28) |
| --- | --- |
|  | (2.29) |
|  | (2.30) |
|  | (2.31) |
|  | (2.32) |
|  | (2.33) |
|  | (2.34) |
|  | (2.35) |
|  | (2.36) |
|  | (2.37) |
|  | (2.38) |
|  | (2.39) |
|  | (2.40) |
|  | (2.41) |
|  | (2.42) |
|  | (2.43) |
|  | (2.44) |
|  | (2.45) |
|  | (2.46) |
|  | (2.47) |
|  | (2.48) |
|  | (2.49) |
|  | (2.50) |
|  | (2.51) |
|  | (2.52) |
|  | (2.53) |

***Additional Buffering in the Mitochondrial Compartment***

To account for additional buffering not attributed to metabolites and substrates in the mitochondrial compartment, equations 2.21, and 2.45 are modified by adding the following terms:

|  | (2.54) |
| --- | --- |
|  | (2.55) |

***Additional Buffering in the Extra-Mitochondrial Compartment***

Both MOPS and EGTA were included in the incubation medium for the conditions simulated in the main paper. Each of these reagents buffer H+s, and EGTA also buffers Ca2+. Moreover, EGTA is primarily dibasic in the pH range simulated. Chemical impurities and cation binding to biological membranes also modulates extra-mitochondrial buffering of cations. To account for these additional buffers in the extra-mitochondrial compartment, Eqs. 2.47, 2.25, 2.41 and 2.48 are modified by adding the following terms:

|  | (2.56) |
| --- | --- |
|  | (2.57) |
|  | (2.58) |
|  | (2.59) |
|  | (2.60) |

*Flux Terms*

The terms representing flux into a given compartment are defined as

|  | (2.61) |
| --- | --- |
|  | (2.62) |
|  | (2.63) |
|  | (2.64) |
|  | (2.65) |

where *Nk* is the number of reactions, *nk* is the stoichiometric coefficient of *k*th reaction, *Jk* is the *k*th reaction rate and is the *i*th cation transport rate into the compartment.

The ligand dependent partial derivative expressions are defined as

|  | (2.66) |
| --- | --- |
|  | (2.67) |
|  | (2.68) |
|  | (2.69) |
|  | (2.70) |

*Compartment Reactions and Fluxes*

The generation of protons by biochemical reactions in the mitochondria is defined as

|  | (2.71) |
| --- | --- |

This term is zero in the extra-mitochondrial compartment.

The transport of cations into the mitochondrial compartment are defined as

|  | (2.72) |
| --- | --- |
|  | (2.73) |
|  | (2.74) |
|  | (2.75) |
|  | (2.76) |

The transport terms for the extra-mitochondrial compartment are the negative of their mitochondrial counterparts.

**S3: Model Rate Equations**

***Lumped Mitochondrial Dehydrogenases***

The generalized biochemical equation for the mitochondrial dehydrogenase is defined as shown below. The equation assumes a lumped model of the TCA cycle which includes pyruvate dehydrogenase, citrate synthase, aconitase, isocitrate dehydrogenase, alpha-ketoglutarate dehydrogenase, succinyl-CoA synthetase, and nucleoside-diphosphate kinase. This version is nearly identical to prior work1 but with added pyruvate and oxaloacetate dependencies.

.

The rate expression used in the model is

.

**Table S3.1.** Mitochondrial Dehydrogenase Parameters

| Parameter | Definition | Value | Reference |
| --- | --- | --- | --- |
| *XDH* | Dehydrogenase activity | 7213 nmol/min/mg | a |
| *KN,DH* | NADH/NAD+ feedback constant | 7.6x10-3 | a |
| *KA,DH* | ATPase feedback constant | 124 µM | a |
| *KPYR,DH* | PYR binding constant | 33 µM | 4 |
| *KOAA,DH* | OAA binding constant | 4 µM | 4 |
| *nDH* | ATPase feedback Hill coefficient | 1.15 | 5 |

a Adjustable parameter fit to data in accompanying article.

***Monocarboxylate Transporter***

The monocarboxylate transporter (MCT) was modeled using the following simple mass-action empirical expression with *VMCT* equal to 3.12 x1013 M-1min-1.

***Dicarboxylate Carrier***

The dicarboxylate carrier (DCC) exchanges TCA cycle intermediates malate, succinate, and hydrogen phosphate. The enzyme reaction mechanism was based on a symmetric, flip-flop type mechanism with competition between all three solutes competing for the enzyme binding site. The principles of mass action and rapid-equilibrium binding was used to derive the following rate equation.

**Table S3.2.** Dicarboxylate Carrier Parameters

| Parameter | Definition | Value | Reference |
| --- | --- | --- | --- |
| *VDCC* | Maximum exchange rate | 6.86 x103 nmol/min/mg | a |
| *KMAL* | MAL binding constant | 2 mM | 1 |
| *KSUC* | SUC binding constant | 1 mM | 1 |
| *KPi* | Pi binding constant | 4.3 mM | 1 |

a Adjustable parameter fit to data in accompanying article.

***Fumarate hydratase***

Fumarate hydratase (FH) is the enzyme responsible for the condensation reaction incorporating a water molecule into fumarate to form malate. We used the reaction mechanism, binding constants and free energies described in Bazil et al.4. For completeness, the rate expression used in the model is presented below.

The reference reaction for FH is defined as

,

the biochemical equation is

and to simplify the notation, the reactants are redefined as follows: [A] = [FUM]m and [P] = [MAL]m.

The apparent equilibrium constant for this reaction is defined as

.

The overall rate expression for FH used in the model is

where the reverse rate is defined using the Haldane constraint as

and

.

**Table S3.3.** Fumarate Hydratase Parameters

| Parameter | Definition | Value | Reference |
| --- | --- | --- | --- |
| *Vmf* | Maximum forward reaction rate | 5.45 x103 nmol/min/mg | a |
|  | Gibbs free energy of reaction | -3.6 kJ/mol | 4 |
| *KmA* | FUM binding constant | 44.7 µM | 4 |
| *KmB* | MAL binding constant | 198 µM | 4 |
| *KiCIT* | CIT inhibition constant | 3.50 mM | 4 |
| *KiATP* | ATP inhibition constant | 40.0 µM | 4 |
| *KiADP* | ADP inhibition constant | 400 µM | 4 |
| *KiGTP* | GTP inhibition constant | 80.0 µM | 4 |
| *KiGDP* | GDP inhibition constant | 330 µM | 4 |

a Fit to simulation using the experimental data sets described in the main paper.

***Malate dehydrogenase***

Malate dehydrogenase (MDH) is the enzyme responsible for the oxidation of malate to form oxaloacetate and reducing equivalents for the ETS. We used the reaction mechanism, binding constants and free energies described in Bazil et al.4. For completeness, the rate expression used in the model is presented below.

The reference reaction for MDH is defined as

,

the biochemical equation is

and to simplify the notation, the reactants are redefined as follows: [A] = [NAD]m, [B] = [MAL]m, [P] = [OAA]m,and [Q] = [NADH]m.

Since the reaction catalyzed by the enzyme MDH involves the generation of a proton, the equilibrium constant for this reaction is defined as

.

The overall rate expression for MDH used in the model is

where the reverse rate is defined using the Haldane constraint as

and

.

**Table S3.4.** Malate Dehydrogenase Parameters

| Parameter | Definition | Value | Reference |
| --- | --- | --- | --- |
| *Vmf* | Maximum forward reaction rate | 965 nmol/min/mg | a |
|  | Gibbs free energy of reaction | 69.13 kJ/mol | 4 |
| *KmA* | NAD binding constant | 90.6 µM | 4 |
| *KmB* | MAL binding constant | 250 µM | 4 |
| *KiA* | NAD inhibition constant | 279 mM | 4 |
| *KiB* | MAL inhibition constant | 360 µM | 4 |
| *KmP* | OAA binding constant | 6.13 µM | 4 |
| *KmQ* | NADH binding constant | 2.58 µM | 4 |
| *KiP* | OAA inhibition constant | 5.50 µM | 4 |
| *KiQ* | NADH inhibition constant | 3.18 µM | 4 |
| *KiATP* | ATP inhibition constant | 183 µM | 4 |
| *KiADP* | ADP inhibition constant | 394 µM | 4 |
| *KiAMP* | AMP inhibition constant | 420.0 µM | 4 |
| *KPi* | Pi binding constant | 5.00 mM | 4 |
| *βPi* | Pi activation constant | 57.4 | 4 |

a Fit to simulation using the experimental data sets described in the main paper.

***Malic Enzyme***

Malic enzyme reaction is modeled using the following simple equation:

This reaction produces pyruvate, NADH, and a CO2 as products. The value of *kfME* is 3.03 x106 M-1min-1 and was fit to data presented in the accompanying article.

***NADH-ubiquinone oxidoreductase: Complex I***

The biochemical equation for Complex I is defined as

.

For full model details, see Bazil et al.14. In brief, the model is a five-state model that includes the minimal components necessary to simulate NADH-quinone oxidoreductase activity as a function of pH and mitochondrial membrane potential (ΔΨ). It includes the detailed redox biochemistry required to simulate ROS generation by both the FMN (IFMN) and SQ (IQ) sites. The model consists of numerous binding polynomials that represent various combinations free, substrate-bound, product-bound, and protonated states and characterizes the multiple redox states of the enzyme. In addition, the thermodynamic equations are corrected for pH, temperature, and ionic strength. The model equations consist of about a hundred supporting equations that culminate into the following matrix equation describing the steady state fractional occupancies of the different redox states:

For definitions of the individual rate constants and their constituents (*kN*’s, *kSO*’s, and *kH*’s), see the original article14. After solving for the state occupancy vector, the rate of NADH oxidation is given as:

.

The rate of superoxide generation is given as:

.

The rate of hydrogen peroxide generation is given as:

.

And for numerical stability, the rate of Q reduction is coupled to the above rates such that:

.

**Table S3.5.** NADH-Ubiquinone Oxidoreductase Parameters

| Parameter | Definition | Value | Reference |
| --- | --- | --- | --- |
| *Adjustable Parameters* | | | |
| *Etot,CI* | Total Complex I content | 167 pmol/mg | a |
| *Krot* | Rotenone binding constant | 1.48 pM | a |
| *Structural and Thermodynamic Parameters* | | | |
|  | FMN/FMNH2 midpoint potential | -368 (55) mV | 15 b |
|  | FMN/FMNH· midpoint potential | -332 (24) mV | 15 b |
|  | FMNH·/FMNH2 midpoint potential | -404 (87) mV | 15 b |
|  | FMNH2 pK | 7.1 | 15 b |
|  | FMNH.- pK | 7.9 | 15 b |
|  | N2ox/N2red midpoint potential | -153 (-90) mV | 16 c |
|  | N2ox pK | 6 | 16 c |
|  | N2red pK | 8.5 | 16 c |
| *Kinetic Parameters* | | | |
|  | NADH dissociation constant for oxidized FMN | 4.61x10-5 M | 1 |
|  | NAD+ dissociation constant for oxidized FMN | 7.05 x10-4 M | 1 |
|  | NADH dissociation constant for reduced FMN | 4.99 x10-4 M | 1 |
|  | NAD+ dissociation constant for reduced FMN | 1.18 x10-5 M | 1 |
|  | NADH dissociation constant for FMN radical | 1 M | 1 |
|  | NAD+ dissociation constant for FMN radical | 1.54 x10-4 M | 1 |
|  | Q10H2 dissociation constant | 1.00 x10-1 M | 1 |
|  | Q10 dissociation constant | 1.75 x10-2 M | 1 |
|  | Q10 stability constant | 10 | 1 |
|  | NADH oxidation rate for state 0 | 117850 min-1 | 1 |
|  | NADH oxidation rate for state 2 | 1.12 x104 min-1 | 1 |
|  | NADH oxidation rate for state 1 | 277 min-1 | 1 |
|  | Q reduction rate for state 2 | 3.49 x105 min-1 | 1 |
|  | Q reduction rate for state 4 | 4.67 x1012 min-1 | 1 |
|  | Q reduction rate for state 3 | 5.20 x102 min-1 | 1 |
| *β* | Charge translocation parameter | 0.5 | 1 |
|  | NADH oxidase site pK | 7.39 | 1 |
|  | Q reductase site pK | 6.41 | 1 |
| *ROS Parameters* | | | |
|  | Superoxide production rate from semiquinone for state 1 | 1.00 x1011 M-1min-1 | 1 |
|  | Superoxide production rate from semiquinone for state 2a | 0.124 M-1min-1 | 1 |
|  | Superoxide production rate from semiquinone for state 2b | 0.124 M-1min-1 | 1 |
|  | Superoxide production rate from semiquinone for state 3a | 6.87 x10-4 M-1min-1 | 1 |
|  | Superoxide production rate from semiquinone for state 3b | 1.44 x10-7 M-1min-1 | 1 |
|  | Superoxide production rate from semiquinone for state 4 | 1.30 x10-5 M-1min-1 | 1 |
|  | Superoxide production rate from FMNH2 for state 2 | 2.71 x108 M-1min-1 | 1 |
|  | Superoxide production rate from FMNH2 for state 3a | 0.033 M-1min-1 | 1 |
|  | Superoxide production rate from FMNH2 for state 3b | 0.017 M-1min-1 | 1 |
|  | Superoxide production rate from FMNH2 for state 4 | 8.41 x106 M-1min-1 | 1 |
|  | Superoxide production rate from FMNH.- for state 1 | 4.08 x109 M-1min-1 | 1 |
|  | Superoxide production rate from FMNH.- for state 2a | 2.43 x10-9 M-1min-1 | 1 |
|  | Superoxide production rate from FMNH.- for state 2b | 7.17 x10-6 M-1min-1 | 1 |
|  | Superoxide production rate from FMNH.- for state 3 | 0.839 M-1min-1 | 1 |
|  | Hydrogen peroxide production rate from FMNH2 for state 2 | 4.69 x10-5 M-1min-1 | 1 |
|  | Hydrogen peroxide production rate from FMNH2 for state 3a | 1.27 x108 M-1min-1 | 1 |
|  | Hydrogen peroxide production rate from FMNH2 for state 3b | 1.06 x108 M-1min-1 | 1 |
|  | Hydrogen peroxide production rate from FMNH2 for state 4 | 3.72 x103 M-1min-1 | 1 |

a Adjustable parameter fit to data in accompanying article. b Refit from data in Figures 1, 2 and 3 of Sled et al. 15 with values in parenthesis corresponding to pH = 0 and T = 25 °C conditions. c Refit from data in Figure 1 of Ingledew and Ohnishi 16.

***Succinate-ubiquinone oxidoreductase: Complex II***

The biochemical equation for Complex II is defined as

.

In brief, the model is a five-state model that includes the minimal components necessary to simulate succinate dehydrogenase activity as a function of pH and includes the detailed redox biochemistry required to simulate ROS generation by the FAD (IIFAD) and Q sites (IIQ). The model consists of numerous binding polynomials that represent various combinations free, substrate-bound, product-bound, and protonated states and characterizes the multiple redox states of the enzyme. In addition, the thermodynamic equations are corrected for pH, temperature, and ionic strength. The model equations consist of about a hundred supporting equations that culminate into the following matrix equation describing the steady state fractional occupancies of the different redox states:

For definitions of the individual rate constants (*kij*) and their constituents, see the original article17. After solving for the state occupancy vector, the rate of SUC oxidation is given as:

| where  and is the OAA-SDH bound complex solved using the binding polynomial method12. |
| --- |

The superoxide formation steady-state rate is given as:

| **.** |
| --- |

The hydrogen peroxide formation steady-state rate is:

| . |
| --- |

And the quinol reduction steady-state rate is:

| . |  |
| --- | --- |

Below is an updated list of parameters.

**Table S3.6.** Complex II Parameters a

| Parameter | Definition | Value | Reference |
| --- | --- | --- | --- |
| *Adjustable Parameters* | | | |
| *Etot,CII* | Succinate dehydrogenase content | 725 nmol/mg | b |
| *Structural and Thermodynamic Parameters* | | | |
|  |  |  |  |
|  | pKa for flavin free radical | 8 | 18 |
|  | pKa for fully reduce flavin | 7.7 | 18 |
|  | FAD/FADH· midpoint potential | -146 (385) mV | 18 c |
|  | FADH·/FADH2 midpoint potential | -41 (284) mV | 18 c |
|  | FAD/FADH2 midpoint potential | -93 (334) mV | 18 c |
|  | Midpoint potential of [*2Fe-2S*]ox,,red | 0 mV | 19 |
|  | Midpoint potential of [*4Fe-4S*] ox,,red | -260 mV | 19 |
|  | Midpoint potential of [*3Fe-4S*] ox,,red | 60 mV | 19 |
|  | O2/O2•- midpoint potential | -160 mV | 20 |
|  | O2/H2O2 midpoint potential | 320 mV | 20,21 |
|  | Q/Q•- midpoint potential | 284 mV | 17 |
|  | atpenin inhibitory factor | 14.6 | 17 |
|  | atpenin dissociation constant at Qp site | 1.67 x 10-4 pM | 17 |
|  | atpenin dissociation constant at Qd site | 68.3 nM | 17 |
| *Kinetic parameters* | | | |
|  | rate constant for SUC oxidation | 3170 min-1 | 17 |
|  | rate constant for QH2 production | 1.49 x109 min-1 | 17 |
|  | SUC dissociation constant | 355 µM | 17 |
|  | FUM dissociation constant | 1.0 mM | 17 |
|  | Q dissociation constant | 0.29 nM | 17 |
|  | QH2 dissociation constant | 0.19 nM | 17 |
|  | H+ dissociation constant at Qp-site | 170 nM | 17 |
|  | malonate dissociation constant | 14.8 µM | 17 |
|  | MAL dissociation constant | 294 µM | 17 |
|  | OAA dissociation constant | 0.822 µM | 17 |
|  | OAA inactivation constant | 0.1 | 17 |
| *ROS Parameters* | | | |
|  | rate constant for O2•- production by FADH• | 4.89 x107 M-1min-1 | 17 |
|  | rate constant for O2•- production by [*3Fe-4S*] | 4.00 x1011 M-1min-1 | 17 |
|  | rate constant for H2O2 production by FADH2 | 1.57 x105 M-1min-1 | 17 |

a Updated from Manhas et al.17 b Adjustable parameter fit to data in accompanying article. c Values in parenthesis are for pH 0 and at 25 °C conditions.

***Ubiquinol-cytochrome-c oxidoreductase: Complex III***

The biochemical equation for Complex III is defined as

.

For model details, see Bazil et al.2. In brief, the model is a six-state model that simulates quinol-cytochrome c oxidoreductase activity as a function of pH and ΔΨ, as well as, the redox biochemistry involved in superoxide formation. Electrons are added or removed from the complex either one at a time at the Qp-site or two at a time at the Qn-site. The last row in the matrix equation given below is a conservation equation to fix the sum of the state occupancies to equal one.

The model consists of numerous binding polynomials that represent various combinations free, substrate-bound, product-bound, and protonated states and characterizes the multiple redox states of the enzyme. In addition, the thermodynamic equations are corrected for pH, temperature, and ionic strength. The model equations consist of dozens of supporting equations that culminate into the following matrix equation describing the steady state fractional occupancies of the different redox states:

For definitions of the individual rate constants, their constituents (e.g., *kQH2c3+*’s, *kQH2*’s, and *kSO*’s), and other model terms, see the original article. After solving for the state occupancy vector, the rate of cytochrome c reduction is given as:

.

The rate of superoxide generation is given as:

.

And for numerical stability, the rate of ubiquinol oxidation is coupled to the above rates such that:

.

**Table S3.7.** Ubiquinol-Cytochrome-c Oxidoreductase Parameters a

| Parameter | Definition | Value | Reference |
| --- | --- | --- | --- |
| *EtotC3* | Total Complex III content | 0.372 pmol/mg | b |
|  | Qp-site stability constant for Q10 | 1.40 x10-12 | b |
| *KA values* | | | |
|  | Oxidized Rieske iron-sulfur cluster acidic group protonation constant | 10-6.6 | 22 |
|  | Oxidized Rieske iron-sulfur cluster basic group protonation constant | 10-9.2 | 22 |
|  | Oxidized heme bL protonation constant | 10-5.9 | 23 |
|  | Reduced heme bL protonation constant | 10-7.9 | 23 |
|  | Oxidized heme bH protonation constant | 10-5.7 | 23 |
|  | Reduced heme bH protonation constant | 10-7.7 | 23 |
| *Thermodynamic values* | | | |
|  | Rieske ISP midpoint potential | 311 mV | 24 |
|  | Cytochrome bL midpoint potential | -33 (39) mV | 23 c |
|  | Cytochrome bH midpoint potential | 84 (160) mV | 23 c |
|  | Qn-site semiquinone midpoint potential | -37 mV | 25 |
|  | Qn-site semiquinone midpoint potential | 92 (954) mV | 25 c |
|  | Fraction of total charge translocation sensed between bL and bH | 0.5 | 23,26-28 |
|  | bH/bL monomeric Coulombic interaction energy | 11.6 kJ/mol | 2 |
|  | bL/bL dimeric Coulombic interaction energy | 5.3 kJ/mol | 2 |
|  | Qp-site stability constant for Q10 | 2.28 x10-15 | 2 |
| *Q10 related constants* | | | |
|  | Q10H2 binding constant at Qp-site | 1.6 mM | 29 |
|  | Q10 binding constant at Qp-site | 1.6 mM | 29 |
|  | Q10 binding constant at Qn-site | 0.25 mM | 30 d |
|  | Q10H2 binding constant at Qn-site | 2.5 µM | 30 d |
| Q10,tot | Q10 pool size | 20 mM | 31,32 e |
| *Superoxide related constants* | | | |
|  | Superoxide production 2nd order rate constant | 1010 M-1min-1 | 33 |
| *Kinetic rate constants* | | | |
|  | Quinol oxidation rate for *E0* | 1.43 x105 min-1 | 2 |
|  | Quinol oxidation rate for *E1* | 7.53 x104 min-1 | 2 |
|  | Quinol oxidation rate for *E2* | 1.89 x105 min-1 | 2 |
|  | Quinol oxidation rate for *E3* | 1.43 x104 min-1 | 2 |
|  | Quinol oxidation rate for *E4* | 248 min-1 | 2 |
|  | Quinone reduction rate for *E2* | 5.52 x1011 min-1 | 2 |
|  | Quinone reduction rate for *E3* | 5.52 x1011 min-1 | 2 |
|  | Quinone reduction rate for *E4* | 4.19 x105 min-1 | 2 |
|  | Quinone reduction rate for *E5* | 6.78 x1011 min-1 | 2 |
| *Cytochrome c binding constants* | | | |
|  | c3+ binding constant | 1.11 x10-6 M | 2 |
|  | c2+ binding constant | 2.49 x10-6 M | 2 |
| a Values updated from Bazil et al.2  b Adjustable parameter fit to data in accompanying article. c Values in parenthesis are at pH 0 and 25 °C. d Values chosen to match reported kinetic and thermodynamic behavior of enzyme. e Estimated by assuming a total mitochondrial Q pool of 5.8 nmol/mg and an inner membrane volume of 290 nl/mg. | | | |

***Cytochrome-c oxidase: Complex IV***

The biochemical equation for Complex IV is defined as

.

The equilibrium constant is defined as

.

The rate expression used in the model is

.

**Table S3.8.** Cytochrome-c Oxidase Parameters

| Parameter | Definition | Value | Reference |
| --- | --- | --- | --- |
| *XC4* | Complex IV activity | 1.2x104 nmol/mg/min | 5 |
|  | Gibb’s free energy of reaction | -202.16 kJ/mol | 34 |
|  | O2 binding constant | 1 µM | 11 |
| *KM* | c2+ binding constant | 162 µM | 35 |
| *n* | Hill coefficient for c2+ | 2 | 35 |
| *β* | ΔΨ constant | 6.6x10-6 | 35 |

***Proton leak***

The electrophoretically driven proton uptake via leak pathways was updated to account for the highly non-linear nature of the leak across energy-transducing membranes 36. XHleak was set to 3.72 x107 nmol/mg/min/M base on prior work5.

.

***Adenine nucleotide translocase (ANT)***

The biochemical equation for ANT is defined as

.

The rate expression used in the model is

,

where

,

,

,

,

and

.

**Table S3.9.** Adenine Nucleotide Translocase Parameters a

| Parameter | Definition | Value | Reference |
| --- | --- | --- | --- |
| *EtotANT* | Total ANT content | 57.2 nmol/mg | 1 |
|  | Forward translocation rate | 636 min-1 | 34 |
|  | Reverse translocation rate | 1.74E+3 min-1 | 34 |
|  | ADP binding constant | 1.5021 x10-005 M | 34 |
|  | ATP binding constant | 1.5021 x10-005 M | 34 |
| *α1* | Translocation displacement constant | 0.2741unitless | 34 |
| *α2* | Translocation displacement constant | 0.1926 unitless | 34 |
| *α3* | Translocation displacement constant | -0.2397 unitless | 34 |
| *δT* | ATP displacement binding constant | 0.0459 unitless | 34 |
| *δD* | ADP displacement binding constant | -0.0088 unitless | 34 |

a Updated from Metelkin et al.37.

***F1FO ATP synthase***

The biochemical equation for F1FO ATP synthase is defined as

.

The equilibrium constant is defined as

.

The rate expression used in the model is

.

**Table S3.10.** F1FO ATP synthase Parameters

| Parameter | Definition | Value | Reference |
| --- | --- | --- | --- |
| *XF1FO* | F1FO activity | 7.5 x1007 nmol/M/min/mg | 11 a |
|  | Gibb’s free energy of reaction | -4.51 kJ/mol | 34 |
| *nH* | H+:ATP ratio | 8/3 | 38 |

a The activity was lowered from the cited value to reduced the stiffness of the system of DAEs. This resulted in negligible differences in state variable dynamics for the simulations that were used to identify this parameter value.

***Inorganic phosphate carrier (PiC)***

The biochemical equation for the PiC is defined as

.

The rate expression used in the model is

.

A is calculated as

P is calculated as

**Table S3.11** Inorganic Phosphate Parameters

| Parameter | Definition | Value | Reference |
| --- | --- | --- | --- |
| *XPiC* | PiC activity | 3.08x1012 nmol/min/mg | 11 |
| *kPiC* | Pi binding constant | 1.61 mM | 11 |

***ATPase***

The biochemical equation for an ATPase is defined as

.

The equilibrium constant is defined as

.

The rate expression used in the model is

.

**Table S3.12.** ATPase Parameters

| Parameter | Definition | Value | Reference |
| --- | --- | --- | --- |
| *XATPase* | ATPase activity | 50 µM/min | a |
|  | Gibb’s free energy of reaction | 4.51 kJ/mol | 34 |
|  | ADP inhibition constant | 262 µM | 39 |

a The value was based the steady state respiration rate after a bolus of ADP (i.e., ATP cycling state).

***Potassium-hydrogen exchanger***

The biochemical equation for KHE is defined as

.

The rate expression used in the model is

where *XKHE* is set to 2.0x1010 mol/min/mg.

***Calcium uniporter***

For the calcium uniporter, we opted to use as simple of a model as possible to explain the data. This model is derived assuming the energy barrier is centered in the membrane with a second allosteric calcium cooperative binding constant equal to the channel binding constant.

The biochemical equation for the MCU is defined as

.

The rate expression used in the model is

**Table S3.13.** Mitochondrial Calcium Uniporter Parameters

| Parameter | Definition | Value | Reference |
| --- | --- | --- | --- |
| *XMCU* | MCU activity | 3.96E+5 nmol/min/mg | 1 |
| *KCa* | Ca2+ binding constant | 4.8 mM | 1 |

a Fit by simulating the experimental data sets described in the main paper.

***Sodium-calcium exchanger***

In order to balance Ca2+ influx from the calcium uniporter, mitochondria utilize what is known as the sodium-calcium exchanger (NCLX). The exchanger stoichiometry is assumed to be 3:1 resulting in an electrogenic exchange of three Na+ for one Ca2+. The expression is based on a rapid equilibrium random bi-bi mechanism and is similar to the Bazil et al. expression4 with no Ca2+-activation.

The biochemical equation for the NCLX is defined as

.

The rate expression used in the model is

.

**Table S3.14.** Sodium-Calcium Exchanger Parameters

| Parameter | Definition | Value | Reference |
| --- | --- | --- | --- |
| *XNCLX* | Sodium/Calcium exchanger activity | 104 nmol/min/mg | 1 |
| *KNa* | Sodium binding constant | 8 mM | 1 |
| *KCa* | Calcium binding constant | 240 µM | 1 |

***Sodium-hydrogen exchanger***

The balance of sodium is achieved via the sodium-hydrogen exchanger. This exchanger is modeled similarly to sodium-calcium exchanger in that it assumes a rapid equilibrium random bi-bi mechanism except for an additional dependence on matrix pH as in Nguyen et al. 40. The proton regulatory binding constant was refit to the data presented in Kapus et al. 41. This rate was modified slightly since a better fit to the data was achieved by including a Hill coefficient of 2 in the regulatory binding term.

The biochemical equation for the NHE is defined as

.

The rate expression for mitochondrial Na+/H+ exchange is,

**Table S3.15.** Sodium-Hydrogen Exchanger Parameters

| Parameter | Definition | Value | Reference |
| --- | --- | --- | --- |
| *XNHE* | Sodium/Hydrogen exchanger | 9.45 x105 nmol/min/mg | 1 |
| *KNa* | Sodium binding constant | 24.3 mM | 1 |
| *KH* | Proton binding constant | 10-8.5 M | 1 |
| *KH,reg* | Proton regulatory binding constant | 10-7.2 M | 1 |

***Manganese Superoxide Dismutase (MnSOD)***

The biochemical equation for MnSOD is defined as

.

The rate expression used in the model is

and

where the subscripts *m* and *e* denote matrix and extra-mitochondrial, respectively, *XSOD* is set to 2.0x104 min-1 based on 10 µM [MnSOD] and a second order rate constant of 2.0x109 M-1min-1 as given in 42,43. For simplicity, the extra-mitochondrial activity was set equal to the mitochondrial activity. In the experiments described in the accompanying article, exogenous SOD was used in excess to prevent accumulation of superoxide in the buffer.

***H2O2 Reduction via Scavenging System***

The biochemical equation for H2O2 scavenging is defined as

.

The rate expression used in the model is

where Vmax,scavenging and KM,H2O2 were fit to data in the accompanying article. The fitted values are 31.6 nmol/min/mg and 106 nM, respectively.

The expression for H2O2 permeation through the mitochondrial membrane is

where *kperm* is 5.5 x106 min-1 based on fitting to the data in the accompanying article.

***Horseradish Peroxidase/Amplex Ultrared Assay***

Lastly, we include the horseradish peroxidase/amplex ultrared assay using the following empirical expression:

Where *kHrP* is set to 106 min-1, an arbitrarily high value to ensure all exported H2O2 is converted to resorufin as expected to occur in the experimental system.

***Parameter Sensitivity Analysis***

The normalized parameter sensitivity matrix is computed using the equation below. Each model output, *fi*, is congruent with the experimental data. The parameter sensitivities were computed using the complex variable approach as described in Squire and Trap 44.

|  |  |
| --- | --- |

The sensitivity coefficients presented in Table 3 of the accompanying article were computed by averaging all the non-zero sensitivity coefficient for a given parameter. This was done by using the following equation.

|  |  |
| --- | --- |

Here, *Ni* is the number of non-zero elements in *i*th row of *S*. This is done to avoid biasing the sensitivity results and to maximize the information obtained from the analysis.

**S4: Supplemental Figures**

|  |
| --- |
| **Figure S1. Malate-dependent respiratory dynamics in the absence and presence of saturating ADP.** The model slightly overpredicts leak state JO2 and slightly underpredicts oxphos JO2. This is due to the simplistic nature of the modeled malic enzyme reaction. These fits are expected to improve with a more rigorous malic enzyme kinetic model including allosteric control mechanisms and a more complete TCA cycle. In these experiments, malate was the sole substrate provided to the mitochondria. |

|  |
| --- |
| **Figure S2.** **Model outputs shown in Fig 11 plotted against the ATPase rate.** Model simulation protocols are described in the legend of Fig 11. A) Respiration rate is linear with respect to the ATPase rate. B) ROS emission rates fall with ATPase rate due to a drain on membrane potential as energy demand increases. C) Membrane potential falls with increasing ATP demand. A strong dependence on the membrane potential for ROS output is reflected in the five-fold drop in ROS emission rates when the membrane potential decreases only about 10% (~20 mV). D) Steady-state buffer ADP concentrations are kept lowest with P/M/S followed by P/M, S, and S/R. The decrease in the respiratory rate with succinate as ATP demand increases is due to OAA inhibition. These profiles plotted versus simulated ATPase rate differs from the Vmax profiles shown in Fig 11 due to the ADP-dependent negative feedback encoded in the expression. Thus, the Vmax does not necessarily reflect the actual activity since different substrates support different energization statuses that are reflected in the sustained ADP levels shown in D). These predictions are consistent with our current understanding of mitochondrial bioenergetics and will be tested in future studies. |

|  |
| --- |
| **Figure S3. Model outputs of redox pools corresponding to conditions described in Fig 11 plotted against the ATPase rate.** Model simulation protocols are described in the legend of Fig 11. The predicted changes of the redox pools NADH (A), QH2 (B), and cyt c2+ (C) as ATP demand increases is shown for each substrate condition used for model calibration and corroboration. The bend in the curves for the succinate condition are due to OAA inhibition as it builds up during an increase in ATP demand. |
|  |
| **Figure S4. Succinate stimulates energy metabolism and ROS emission.** Model simulation protocols were like those used for simulating the experimental conditions presented in the accompanying article. The only major differences are the inclusion of 10 mM ATP in the extramitochondrial space as an initial condition, 0.1 mM pyruvate, 0.5 mM malate, 20 µM O2, and an ATPase activity corresponding to 0.1 mM/min (~50% of max as seen from Fig 11). In these simulations, external metabolites, buffer pH (7.1), and O2 (20 µM) were clamped. The model was then run out to a steady state at the extramitochondrial succinate concentration as shown on the x-axis. These simulation results are consistent with what we know about the impact of succinate metabolism on respiration (A), ROS output (B), membrane potential (C), and extra-mitochondrial, steady-state ADP levels (D), % reduced redox pools (E), and overall electron input contributions to the ETS from NADH (MitoDH, MDH, ME) and QH2 (SDH) producing reactions for the given conditions (F). |

**S5: Model Codes Description**

Generate_Figures.m – mfile used to reproduce the figures in the article

ETS_ROS_model.m – mfile of the system of differential algebraic equations governing model behavior including individual flux models for individual biochemical reactions

data.mat – mat file containing experimental data structure

parameters.mat – mat file containing model parameter structure

RESULTS.mat – mat file containing pre-ran model simulation results

rng_ii.mat – mat file containing random number selection for 4D plots shown in article

**References**

1 Malyala, S., Zhang, Y., Strubbe, J. O. & Bazil, J. N. Calcium phosphate precipitation inhibits mitochondrial energy metabolism. *PLoS Comput Biol* **15**, e1006719, doi:10.1371/journal.pcbi.1006719 (2019).

2 Bazil, J. N. Analysis of a Functional Dimer Model of Ubiquinol Cytochrome c Oxidoreductase. *Biophys J* **113**, 1599-1612, doi:10.1016/j.bpj.2017.08.018 (2017).

3 Bazil, J. N., Blomeyer, C. A., Pradhan, R. K., Camara, A. K. & Dash, R. K. Modeling the calcium sequestration system in isolated guinea pig cardiac mitochondria. *J Bioenerg Biomembr* **45**, 177-188, doi:10.1007/s10863-012-9488-2 (2013).

4 Bazil, J. N., Buzzard, G. T. & Rundell, A. E. Modeling mitochondrial bioenergetics with integrated volume dynamics. *PLoS Comput Biol* **6**, e1000632, doi:10.1371/journal.pcbi.1000632 (2010).

5 Bazil, J. N., Beard, D. A. & Vinnakota, K. C. Catalytic Coupling of Oxidative Phosphorylation, ATP Demand, and Reactive Oxygen Species Generation. *Biophys J* **110**, 962-971, doi:10.1016/j.bpj.2015.09.036 (2016).

6 Smith, R. M., Martell, A. E. & Chen, Y. Critical-Evaluation of Stability-Constants for Nucleotide Complexes with Protons and Metal-Ions and the Accompanying Enthalpy Changes. *Pure Appl Chem* **63**, 1015-1080, doi:DOI 10.1351/pac199163071015 (1991).

7 Smith, R. M. & Alberty, R. A. The Apparent Stability Constants of Ionic Complexes of Various Adenosine Phosphates with Monovalent Cations. *J Phys Chem-Us* **60**, 180-184, doi:DOI 10.1021/j150536a010 (1956).

8 O'Sullivan, W. J. & Smithers, G. W. Stability constants for biologically important metal-ligand complexes. *Methods Enzymol* **63**, 294-336 (1979).

9 Qi, F., Chen, X. & Beard, D. A. Detailed kinetics and regulation of mammalian NAD-linked isocitrate dehydrogenase. *Biochim Biophys Acta* **1784**, 1641-1651, doi:10.1016/j.bbapap.2008.07.001 (2008).

10 Magnus, G. & Keizer, J. Minimal model of beta-cell mitochondrial Ca2+ handling. *Am J Physiol* **273**, C717-733, doi:10.1152/ajpcell.1997.273.2.C717 (1997).

11 Beard, D. A. A biophysical model of the mitochondrial respiratory system and oxidative phosphorylation. *PLoS Comput Biol* **1**, e36, doi:10.1371/journal.pcbi.0010036 (2005).

12 Vinnakota, K. C., Wu, F., Kushmerick, M. J. & Beard, D. A. Multiple ion binding equilibria, reaction kinetics, and thermodynamics in dynamic models of biochemical pathways. *Methods Enzymol* **454**, 29-68, doi:10.1016/S0076-6879(08)03802-0 (2009).

13 Alberty, R. A. *Thermodynamics of biochemical reactions*. (Wiley-Interscience, 2003).

14 Bazil, J. N., Pannala, V. R., Dash, R. K. & Beard, D. A. Determining the origins of superoxide and hydrogen peroxide in the mammalian NADH:ubiquinone oxidoreductase. *Free radical biology & medicine* **77**, 121-129, doi:10.1016/j.freeradbiomed.2014.08.023 (2014).

15 Sled, V. D., Rudnitzky, N. I., Hatefi, Y. & Ohnishi, T. Thermodynamic analysis of flavin in mitochondrial NADH:ubiquinone oxidoreductase (complex I). *Biochemistry* **33**, 10069-10075 (1994).

16 Ingledew, W. J. & Ohnishi, T. An analysis of some thermodynamic properties of iron-sulphur centres in site I of mitochondria. *The Biochemical journal* **186**, 111-117 (1980).

17 Manhas, N. *et al.* Computationally modeling mammalian succinate dehydrogenase kinetics identifies the origins and primary determinants of ROS production. *J Biol Chem* **295**, 15262-15279, doi:10.1074/jbc.RA120.014483 (2020).

18 Tomoko Ohnishi *et al.* Thermodynamic and Electron Paramagnetic Resonance Characterization of Flavin in Succinate Dehydrogena. *J Biol Chem* **256**, 5577-5582 (1981).

19 Grivennikova, V. G., Kozlovsky, V. S. & Vinogradov, A. D. Respiratory complex II: ROS production and the kinetics of ubiquinone reduction. *Biochim Biophys Acta Bioenerg* **1858**, 109-117, doi:10.1016/j.bbabio.2016.10.008 (2017).

20 Mailloux, R. J. Teaching the fundamentals of electron transfer reactions in mitochondria and the production and detection of reactive oxygen species. *Redox Biol* **4**, 381-398, doi:10.1016/j.redox.2015.02.001 (2015).

21 Koppenol, W. H., Stanbury, D. M. & Bounds, P. L. Electrode potentials of partially reduced oxygen species, from dioxygen to water. *Free radical biology & medicine* **49**, 317-322, doi:10.1016/j.freeradbiomed.2010.04.011 (2010).

22 Brandt, U. & Okun, J. G. Role of deprotonation events in ubihydroquinone:cytochrome c oxidoreductase from bovine heart and yeast mitochondria. *Biochemistry* **36**, 11234-11240, doi:10.1021/bi970968g (1997).

23 Rich, P. R., Jeal, A. E., Madgwick, S. A. & Moody, A. J. Inhibitor effects on redox-linked protonations of the b haems of the mitochondrial bc1 complex. *Biochim Biophys Acta* **1018**, 29-40 (1990).

24 Crofts, A. R., Shinkarev, V. P., Kolling, D. R. & Hong, S. The modified Q-cycle explains the apparent mismatch between the kinetics of reduction of cytochromes c1 and bH in the bc1 complex. *J Biol Chem* **278**, 36191-36201, doi:10.1074/jbc.M305461200 (2003).

25 Ohnishi, T. & Trumpower, B. L. Differential effects of antimycin on ubisemiquinone bound in different environments in isolated succinate . cytochrome c reductase complex. *J Biol Chem* **255**, 3278-3284 (1980).

26 Glaser, E. G., Meinhardt, S. W. & Crofts, A. R. Reduction of cytochrome b-561 through the antimycin-sensitive site of the ubiquinol-cytochrome c2 oxidoreductase complex of Rhodopseudomonas sphaeroides. *FEBS letters* **178**, 336-342 (1984).

27 Glaser, E. G. & Crofts, A. R. A new electrogenic step in the ubiquinol:cytochrome c2 oxidoreductase complex of Rhodopseudomonas sphaeroides. *Biochim Biophys Acta* **766**, 322-333 (1984).

28 Robertson, D. E. & Dutton, P. L. The nature and magnitude of the charge-separation reactions of ubiquinol cytochrome c2 oxidoreductase. *Biochim Biophys Acta* **935**, 273-291 (1988).

29 Ding, H. *et al.* Ubiquinone pair in the Qo site central to the primary energy conversion reactions of cytochrome bc1 complex. *Biochemistry* **34**, 15979-15996 (1995).

30 Wikström, M. & Royal Society of Chemistry (Great Britain). *Biophysical and structural aspects of bioenergetics*. (Royal Society of Chemistry, 2005).

31 Lass, A., Agarwal, S. & Sohal, R. S. Mitochondrial ubiquinone homologues, superoxide radical generation, and longevity in different mammalian species. *J Biol Chem* **272**, 19199-19204 (1997).

32 Schwerzmann, K., Cruz-Orive, L. M., Eggman, R., Sanger, A. & Weibel, E. R. Molecular architecture of the inner membrane of mitochondria from rat liver: a combined biochemical and stereological study. *J Cell Biol* **102**, 97-103 (1986).

33 Kuo-chen, C. & Shou-ping, J. Studies on the rate of diffusion-controlled reactions of enzymes. Spatial factor and force field factor. *Sci Sin* **27**, 664-680 (1974).

34 Wu, F., Zhang, E. Y., Zhang, J., Bache, R. J. & Beard, D. A. Phosphate metabolite concentrations and ATP hydrolysis potential in normal and ischaemic hearts. *The Journal of physiology* **586**, 4193-4208, doi:10.1113/jphysiol.2008.154732 (2008).

35 Murphy, M. P. & Brand, M. D. The control of electron flux through cytochrome oxidase. *The Biochemical journal* **243**, 499-505 (1987).

36 Garlid, K. D., DiResta, D. J., Beavis, A. D. & Martin, W. H. On the mechanism by which dicyclohexylcarbodiimide and quinine inhibit K+ transport in rat liver mitochondria. *J Biol Chem* **261**, 1529-1535 (1986).

37 Metelkin, E., Goryanin, I. & Demin, O. Mathematical modeling of mitochondrial adenine nucleotide translocase. *Biophys J* **90**, 423-432, doi:10.1529/biophysj.105.061986 (2006).

38 Nicholls, D. G. & Ferguson, S. J. *Bioenergetics*. Fourth edition / edn, (Academic Press, Elsevier, 2013).

39 Vinnakota, K. C., Bazil, J. N., Van den Bergh, F., Wiseman, R. W. & Beard, D. A. Feedback Regulation and Time Hierarchy of Oxidative Phosphorylation in Cardiac Mitochondria. *Biophys J* **110**, 972-980, doi:10.1016/j.bpj.2016.01.003 (2016).

40 Nguyen, M. H., Dudycha, S. J. & Jafri, M. S. Effect of Ca2+ on cardiac mitochondrial energy production is modulated by Na+ and H+ dynamics. *Am J Physiol Cell Physiol* **292**, C2004-2020 (2007).

41 Kapus, A., Ligeti, E. & Fonyo, A. Na+/H+ exchange in mitochondria as monitored by BCECF fluorescence. *FEBS letters* **251**, 49-52 (1989).

42 Murphy, M. P. Mitochondrial thiols in antioxidant protection and redox signaling: distinct roles for glutathionylation and other thiol modifications. *Antioxidants & redox signaling* **16**, 476-495, doi:10.1089/ars.2011.4289 (2012).

43 Murphy, M. P. How mitochondria produce reactive oxygen species. *The Biochemical journal* **417**, 1-13, doi:10.1042/BJ20081386 (2009).

44 Squire, W. & G. Trapp. Using complex variables to estimate derivatives of real functions. *Siam Rev* **40**, 110-112 (1998).
